# Predicting climate-driven shifts in the breeding phenology of Varied Tits (*Sittiparus various*) in South Korean forests

**DOI:** 10.1080/19768354.2019.1675759

**Published:** 2019-10-10

**Authors:** Min-Su Jeong, Chang-Young Choi, Hankyu Kim, Woo-Shin Lee

**Affiliations:** a Department of Forest Sciences, Seoul National University, Seoul, Republic of Korea; b Research Institute of Agriculture and Life Sciences, Seoul National University, Seoul, Republic of Korea; c Department of Forest Ecosystems and Society, Oregon State University, Corvallis, OR, USA

**Keywords:** Breeding phenology, climate change, egg-laying date, insectivorous songbirds, forest ecosystem

## Abstract

Phenological shifts of plants and animals due to climate change can vary among regions and species, requiring study of local ecosystems to understand specific impacts. The reproductive timing of insectivorous songbirds in temperate forests is tightly synchronized with peak prey abundance, and thus they can be susceptible to such shift in timing. We aimed to investigate the effect of future climate change on the egg-laying phenology of the Varied Tit (*Sittiparus various*), which is common and widely distributed in South Korean forests. We developed the predictive model by investigating their egg-laying dates in response to spring temperatures along geographical gradients, and our model indicated that the tits lay eggs earlier when the average of daily mean and daily maximum temperatures rise. We predicted future shifts in egg-laying dates based on the most recent climate change model published by the Intergovernmental Panel on Climate Change (IPCC), under a scenario with no climate change mitigation and under a scenario with moderate mitigation. Under this outcome, this species might be unable to adapt to rapid climate change due to asynchrony with prey species during the reproductive period. If no mitigation is undertaken, our model predicts that egg-laying dates will be advanced by more than 10 days compared to the present in 83.58% of South Korea. However, even moderate mitigation will arrest this phenomenon and maintain present egg-laying dates. These results demonstrate the first quantitative assessment for the effect of warming temperatures on the phenological response of insectivorous songbirds in South Korea.

## Introduction

Anthropogenic climate change is one of the greatest threats to ecosystems across the globe, introducing a dramatic shift in thermal conditions that organisms and ecological communities have not experienced during their evolutionary history (Harrington et al. 1999; Walther et al. 2002; Parmesan and Yohe 2003). Phenological processes may be among the most sensitive to climate change (Badeck et al. 2004; Edwards and Richardson 2004), with effects occurring at all trophic levels including plant flowering and growing, emergence of arthropods, and breeding of vertebrates (Visser et al. 2006; Burgess et al. 2018; Bell et al. 2019). These shifts in phenology can negatively impact ecological services to local societies and functional interactions in ecosystems across the world (Price 2002; Stenseth and Mysterud 2002; Both et al. 2006). According to current Intergovernmental Panel on Climate Change (IPCC) predictions, global temperature is likely to rise more than 1.5°C above the pre-industrial level by 2040 (IPCC 2018), raising concern for impacts on ecosystem functions.

The timing of reproduction in birds influences reproductive success and adult survival in many bird species (Perrins 1970; Verhulst et al. 1995; Thomas 2001; Visser et al. 2006). Nest-building, incubation, rearing, and other processes incur energetic costs, requiring access to food resources to meet this need (Lack 1968). In many temperate deciduous forests, sufficient caterpillar prey for feeding chicks may be available only for a short period in spring before trees develop defensive mechanisms against herbivory (Feeny 1970; Balen 1973). Insectivorous songbirds must time their egg-laying in order to access this resource during the subsequent rearing period. Therefore, synchrony between songbird breeding and caterpillar biomass is strongly associated with songbird reproductive success (Naef-Daenzer and Keller 1999; Sanz et al. 2003; Visser et al. 2006).

Seasonal breeding of songbird was primarily controlled by the annual change in day length, photoperiod (Dawson et al. 2001; Sharp 2005), but they also use the supplementary cues because the peak timing of caterpillar biomass is highly variable by local environment change (Wingfield et al. 1992; Dawson 2008). The ambient thermal condition is the most important supplement cue (Visser et al. 2009; Schaper et al. 2012), and previous studies have shown that average early spring temperature prior to egg-laying period are correlated with egg-laying dates (Winkel and Hudde 1997; McCleery and Perrins 1998; Visser et al. 2003, 2009; Drake and Martin 2018). On the other hands, captive Great Tits (*Parus major*) respond to both increased temperature and patterns in temperature increment to initiate breeding (Schaper et al. 2012). The response in avian breeding phenology to warming climate has been also observed across the world: for example, Tree Swallows (*Tachycineta bicolor*), Great Tits (*Parus major*) and Pied Flycatcher (*Ficedula hypoleuca*) (Dunn and Winkler 1999; Visser et al. 2003; Both et al. 2004; Both and te Marvelde 2007). However, the degree of the response varied from study to study, reflecting variation between local environment, species, and population processes (Dunn and Winkler 1999; Visser et al. 2003; Both et al. 2004; Both and te Marvelde 2007).

Climate change scenarios project future climate based on greenhouse gas (GHG) emission and socioeconomic factors such as land use and population growth, and are used for risk assessment and development of mitigation strategies (Kim et al. 2013, 2015) and the Representative Concentration Pathway (RCP) scenarios introduced by the IPCC for its fifth Assessment Report (AR5; IPCC 2014). According to meteorological observation for South Korea, spring temperature has risen 2.4°C over the past 106 years (1912–2017; National Institute of Meteorological Sciences 2017) and the climate predictions for the Korean Peninsula from 2071 to 2100 indicate that temperature here will rise more quickly than the global mean (Korea Meteorological Administration 2014). Based on this, the phenology of insectivorous songbirds breeding in South Korean forests will likely shift progressively earlier. However, it has yet to be investigated because of the lack of long-term data on breeding phenology of the birds and regional temperature across South Korea. Temperature patterns along altitude and latitude gradients are often used to study responses to warming and to predict the effect of warming temperature on species distributions (Fielding et al. 2002; Pearson and Dawson 2003), and we adopted this approach in order to examine the predicted outcomes.

We investigated songbird breeding phenology in South Korea in response to warming spring temperatures by comparing models of egg-laying dates with indices of spring temperature prior to the breeding period in Varied Tits (*Sittiparus various*), a common and widely distributed forest species (Lee et al. 2015). Using our model, we predicted how climate change will affect egg-laying dates under two different scenarios, where no GHG emissions mitigation is applied and where moderate mitigation is applied.

## Methods

### Study species

Varied Tits in the family Paridae are cavity-nesting birds preying on insects during the breeding season, distributed across the Korea Peninsula and Japanese Archipelago (Gosler et al. 2019). They are common and abundant in forested habitats in South Korea, and readily use artificial nest boxes, making them useful for studying breeding ecology.

### Study sites and field survey

**Figure 1.**
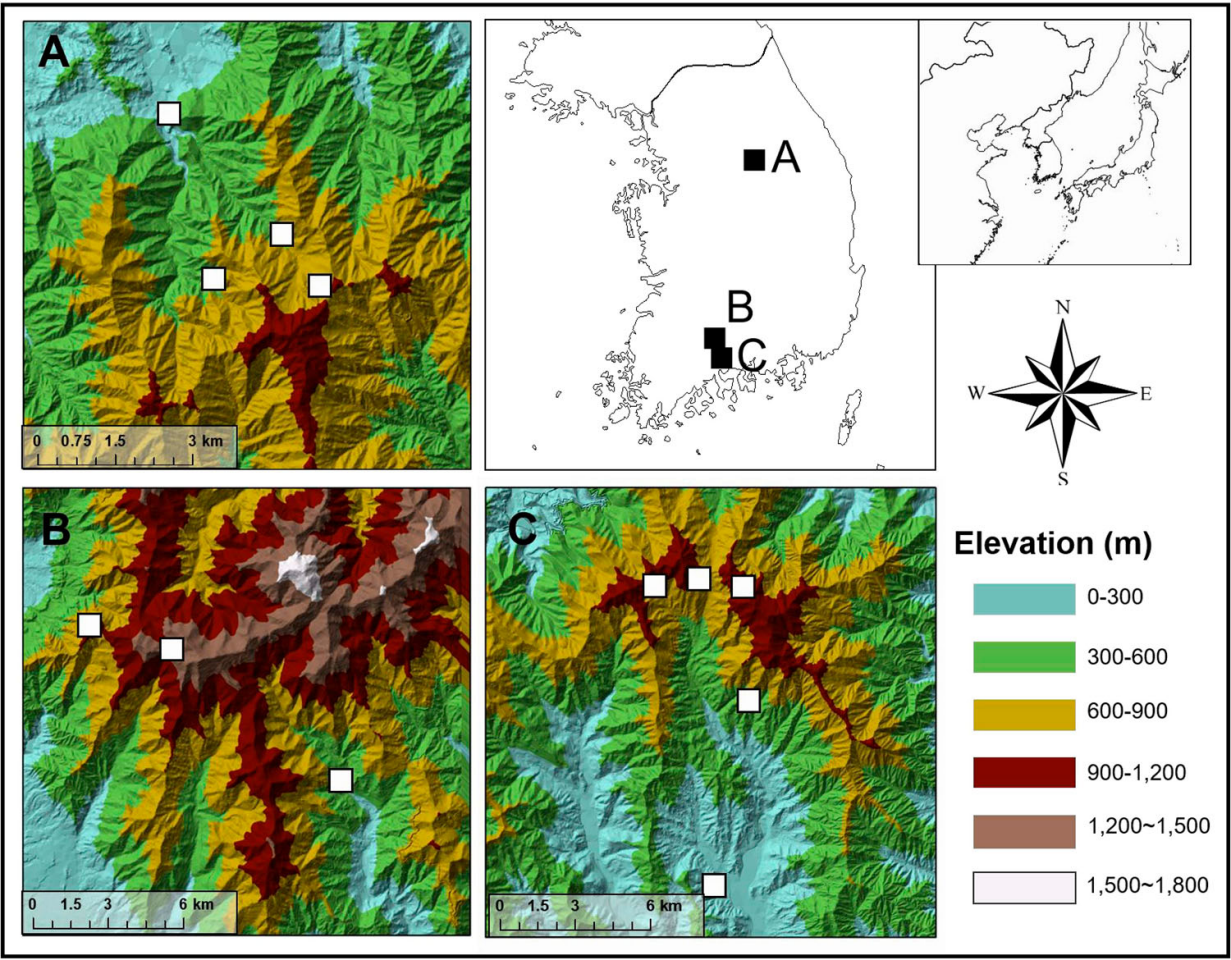
Location of study plots (open squares) in three mountain areas (filled squares): (A) Wonju (B) Gurye and (C) Gwangyang.

Breeding performance data for Varied Tits were collated from 12 study plots in three mountain areas (Gurye, Gwangyang, and Wonju) in South Korea (Figure 1), with the earliest data obtained in 2008 in the two plots in Jirisan Mountains in Gureye-gun (Gurye) and other plots providing data from 2016 to 2018. All plots were located in mixed deciduous oak (*Quercus* spp.) stands reflecting local altitudinal gradients for each mountain system (Figure 1). Each plot was approximately 2 ha in area and located at least 1 km from other plots. Banded individuals were present in plot, and no marked bird was sighted outside of its home plot during a single breeding season. In addition, Great Tits, a confamilial sympatric species with similar ecological traits, has a territory size of roughly 0.67 ha in forest habitats during its breeding season, with timing of its breeding responding to environmental covariates on a fine spatial scale (Hinks et al. 2015). Given these facts, we assumed annual breeding independence between our plots.

We measured local ambient air temperature data of each plot using a temperature-humidity logger (HOBO® pro v2 U23-001, Onset Computer Corporation®, Bourne, USA) with solar radiation shields (RS1, Onset Computer Corporation®), set 2-3 m above the ground at the center of each plot. The observed egg-laying dates of each year and site were from April 7-May 17 and according to previous studies, the temperature about a month before egg-laying season was correlated with egg-laying dates (Winkel and Hudde 1997; McCleery and Perrins 1998). Therefore, the temperature data from March 10–31 of each study year were used to calculate averages of daily mean (mean temperature), daily maximum (max temperature), and rate of increase (temperature slope; the coefficient of the linear regression between date and daily mean temperatures). These indices were selected based on the previous studies about the relationship between the temperature and egg-laying dates of Paridae species (Visser et al. 2003; Schaper et al. 2012; Drake and Martin 2018).

At each site, 48 nest boxes were placed in trees in a 6 × 8 grid or 4 × 12 grid approximately 20–25 m apart, about 1.5 m above the ground. A miniaturized temperature data logger (TDL; iButton DS1291G, Maxim Integrated, San Jose, USA) was installed at the bottom of each nest box and set to record temperatures at intervals of 45 min, to record the changes in thermal signatures of the nest-box by breeding activities. The interior temperature of an occupied, active nest is higher than the ambient temperature or empty nest-boxes, and shows a specific pattern of increase and decrease as the chicks are reared and fledge (Fu et al. 2012; Jeong et al. unpublished data). We compared the temperature of nest-boxes, where breeding of Varied Tit was confirmed, with the average of empty nest-boxes of each year and plot, and then this information was used to estimate breeding timing and eventually the egg-laying dates. Between mid-March and the end of July, nest boxes were regularly visited once a week at five plots in Gwangyang, and other plots were surveyed at least once every 20 days. During these visits, temperature loggers were maintained and breeding stage (nesting, laying, incubation, rearing, or fledging) was recorded, as were the age of chicks. This information was used to support breeding information obtained based on the TDL, such as in the case of malfunction or loss. In these instances, egg-laying dates were estimated by back-counting from hatching dates estimated based on the age of chicks to laying, to the previously known egg incubation period of 13 days (Park 2014). A total of 461 breeding attempts of Varied Tits were recorded from 2008 to 2018, and we included only 364 nests initiated (i.e. first egg laid) within 30 days from the first egg-laying date of the year at each plot, in order to exclude second-clutch nesting events (Bourgault et al. 2010).

### Statistical analysis

We split the egg-laying dates of Varied Tits into training data to construct the predictive model and test data to evaluate the predictive model. Training data is egg-laying dates of 242 nests and climate variables in 12 study plots from 2016 to 2018 and test data is 122 nests and climate variables in two plots of Jiri mountain in 7 years from 2008 to 2015 except 2014.

We used the linear mixed effects modeling approach to develop the predictive model and individual egg-laying dates of training data as response variable (*n* = 242). We compared the three candidate models which included different climate variables (mean temperature, max temperature, and temperature slope) as fixed effect, and a null model to choose the best model with a single variable. In addition, all models included plot and year as random effects, because individual nests are nested within each plot and each year, thus the samples are correlated within year and plot. Heteroscedasticity and the normality of the residuals of models were visually assessed from the residual plots, and we did not observe any violations of model assumptions. We estimated the effect size and 95% confidence interval (CI) of fixed effect in each model.

We used Akaike’s information criterion adjusted for small sample size (AICc) to assess each fixed effect’s explanatory performance in each model and selected model with ΔAICc < 2 as best (Burnham and Anderson 2002). In addition, Akaike weight were used to assess the probability that the model is the best among the candidate models (Burnham and Anderson 2002). We developed a final model of egg-laying dates through conducting model averaging across the identified best models to account for uncertainty of model selection (Burnham and Anderson 2002). We estimated the model averaged effect size, standard error, 85% CI, and 95% CI of fixed effects of final model, because AICc model selection supports variables without overlapping 85% CI (Arnold 2010).

To evaluate the predictive performance of the averaged models for egg-laying dates, we used the temporally independent test data from two plots in the Gurye area for 7 years (*n* = 122). Because the temperature was measured for each plot, we predicted egg-laying dates by plot and year and compared with the average of egg-laying dates by plot and year (*n* = 14). We calculated mean bias error (MBE), root mean square error (RMSE), Pearson correlation coefficient, and R^2^ for the predicted and observed egg-laying dates. In addition, we checked whether the predictive model is overfitting on training data by comparing RMSE and MBE for testing data and training data. To calculate the RMSE and MBE for training data, we calculated the average egg-laying dates by year and plot and compared with the predicted egg-laying dates by using training data (*n* = 36).

We used the projected daily temperature extracted from RCP 8.5 and 4.5 climate scenarios for South Korea with 1 km resolution (Korea Meteorological Administration) to demonstrate the effects of climate change on the egg-laying phenology of Varied Tits. RCP 8.5 represents a scenario with no additional efforts to constrain GHG emission (hereafter referred to as the baseline scenario), with radiative forcing by GHG emission of approximately 8.5 W/m^2^ by 2100 (Riahi et al. 2011). RCP 4.5 represents a scenario with moderate mitigation of GHG emissions using such as reforestation programs, wherein radiative forcing stabilizes at 4.5 W/m^2^ by 2100 (Smith and Wigley 2006; Clarke et al. 2007; Wise et al. 2009). We predicted average egg-laying dates for each 1 km cell for the future periods of 2041–2050, 2071–2080, and 2091–2100. We also calculated the difference of average egg-laying dates between the current decade (2011–2020) and these future periods to show the spatial magnitude of predicted changes across South Korea. Because there are no nationwide egg-laying data for the whole of South Korea, we used the predicted egg-laying dates of the 2010s as present comparators for our future predictions. All statistical procedures were conducted using R 3.5.1 (R Core Team 2018), with *lme4* (Bates et al. 2018) and *MuMIn* (Barton 2019) packages for linear mixed modeling and model selection procedures and *raster* and *rgdal* packages (Bivand et al. 2019; Hijmans 2019) for spatial prediction and mapping egg-laying dates with climate scenarios.

## Results

**Figure 2.**
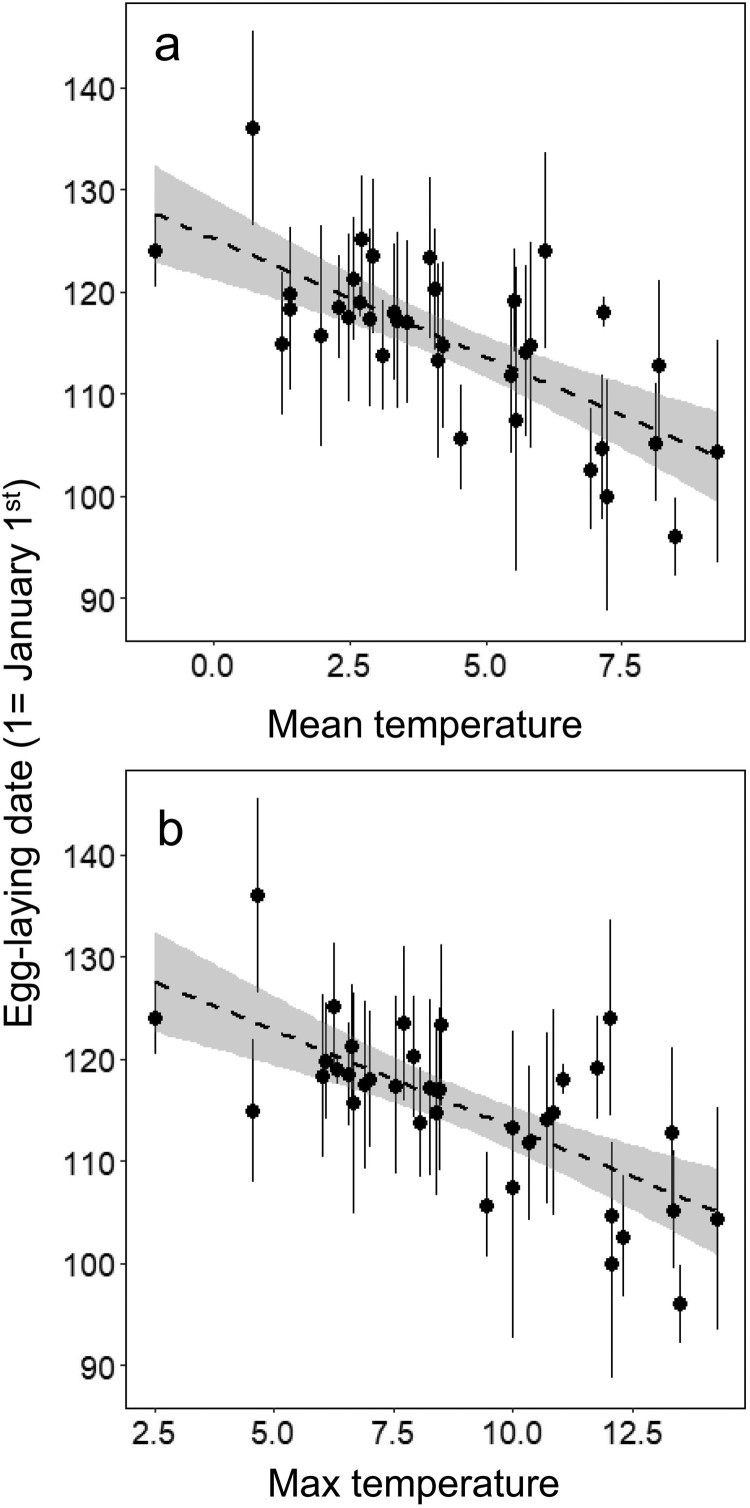
Relationship between (a) mean temperatures and (b) max temperature during the pre-breeding period of March 10–31, and the observed egg-laying dates of Varied Tits (*Sittiparus various*) in South Korea. The dashed line and gray shading represent the regression line and 95% confidence interval.

The mean temperature model ranked highest for explaining variation in observed egg-laying dates and Akaike weight of the model indicated that this model has 73% probability for being the best model (Table 1), followed by the max temperature model (Table 1). The first best model indicated that the egg-laying dates advanced 2.42 days when mean temperatures increased by 1°C (Figure 2(a), Table 1), while the dates advanced 1.95 days as the maximum temperatures increased by 1°C in the second-best model (Figure 2(b), Table 1).

**Table 1.** Results of model comparison based on Akaike’s information criterion adjusted for small sample size (AICc) value and effect size at 95% confidence interval (CI) of the fixed effect of each candidate model.

Variable	Effect size (95% CI)	AICc	ΔAICc	Weight
**Mean temperature**	**−2.42 (−3.29 – −1.54)**	**1728**.**2**	**0**.**00**	**0**.**73**
**Max temperature**	**−1.95 (–2.71 – −1.18)**	**1730**.**1**	**1**.**94**	**0**.**27**
Null	–	1745.8	17.59	0.00
Slope	2.72 (111.55 – 120.19)	1747.7	19.50	0.00

Notes: All candidate models included year and site as random effects. The two compatible best models with ΔAICc < 2.00 in bold were averaged.

**Figure 3.**
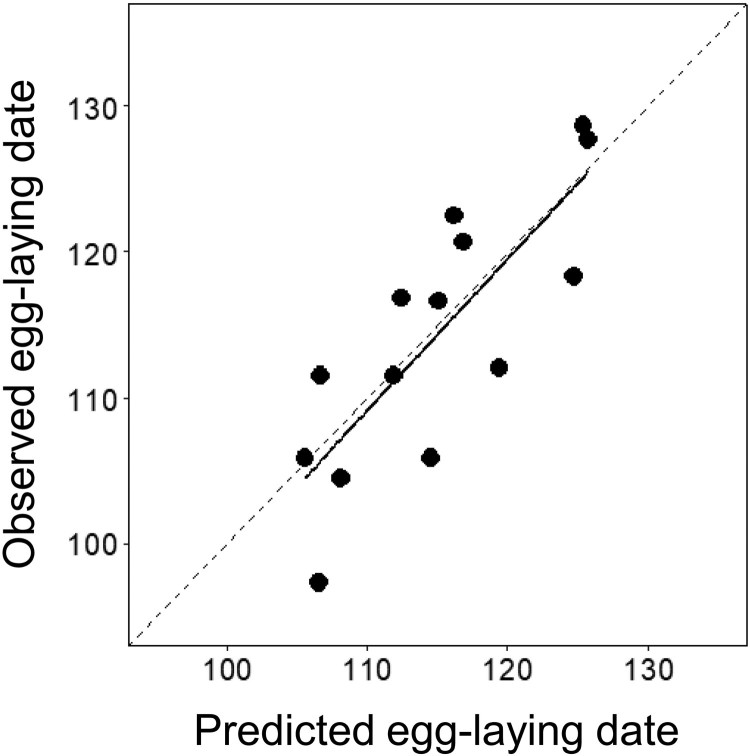
Comparison of observed and predicted mean egg-laying dates of Varied Tits (*Sittiparus various*) in Gurye from 2008 to 2015 using the final averaged model. The solid line represents the regression between the predicted and observed egg-laying dates (where January 1 = 1), and the dashed diagonal line indicates a 1:1 relationship.

The parameter estimates and 85% confidence interval of the final averaged model showed that the egg-laying dates were increased mainly by mean temperature, while both 85% and 95% CI of max temperature did not clearly affect the egg-laying dates by including the zero in their CI ranges (Table 2). As a result of the evaluation of the predictive performance of the final averaged model, the Pearson correlation coefficient was 0.80, indicating a strong positive correlation between predicted and observed egg-laying dates (Figure 3). The RMSE was 5.21, indicating that the average prediction error for egg-laying dates was 5.21 days and the explained variance of the egg-laying dates (R^2^) was 65% (Figure 3). The average model bias (MBE) was 0.64, indicating that the predicted egg-laying dates were overestimated by 0.64 days. In addition, The RMSE and MBE for training data was 5.60 and 0.35, respectively and similar with test data, indicating the predictive model generalize well to temporally independent data.

**Table 2.** Model-averaged effect size and 85% and 95% confidence intervals (CI) for temperature variables affecting egg-laying dates of Varied Tits (*Sittiparus various*).

Variables	Average effect size	85% CI	95% CI
Intercept	127.96 ± 3.98	122.23 – 133.68	120.15 – 135.76
Mean temperature	−1.76 ± 1.13	−3.39 – −0.13	−3.97 – 0.46
Max temperature	−0.53 ± 0.89	−1.81 – 0.74	–2.27 – 1.20

**Figure 4.**
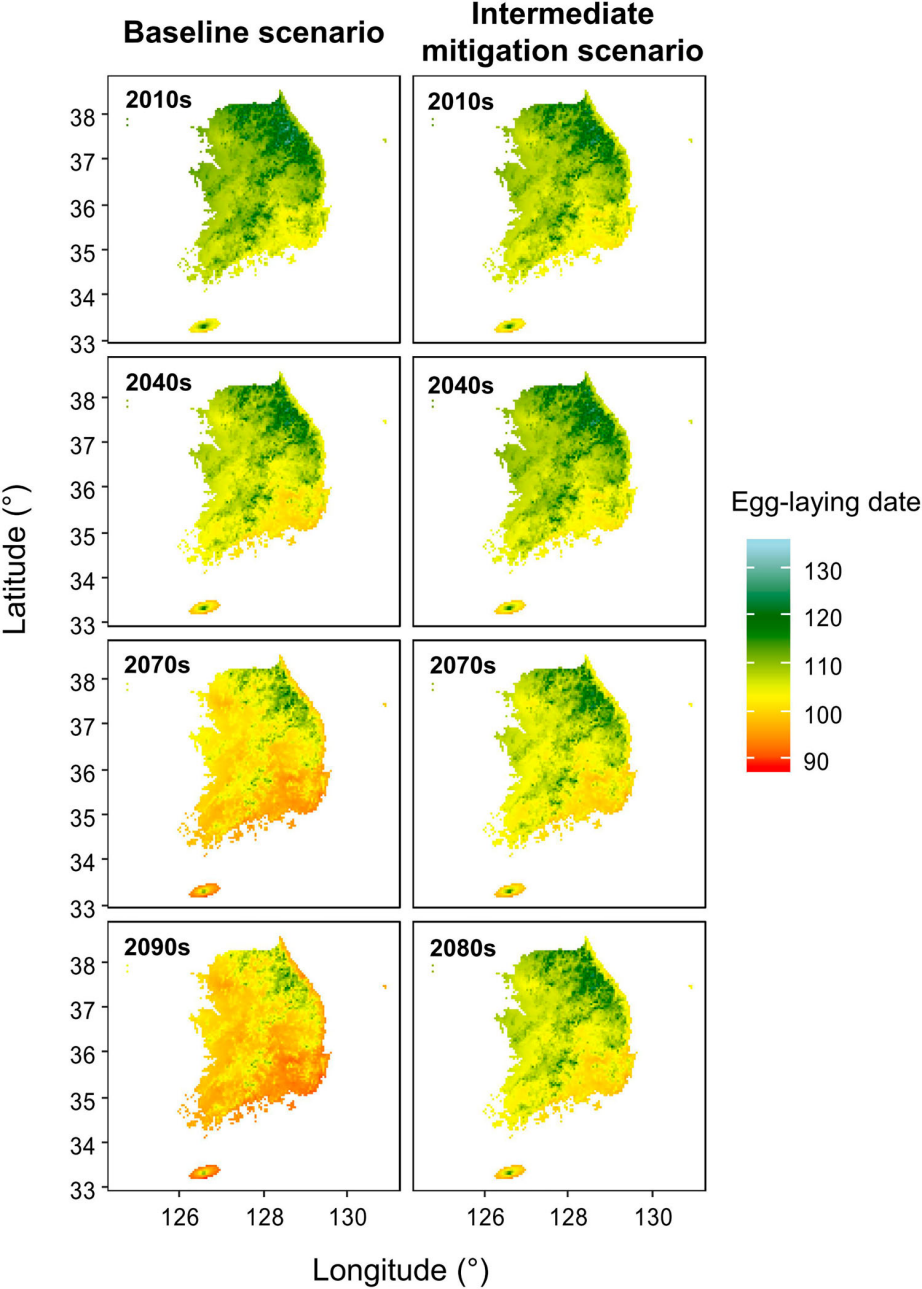
Predicted egg-laying dates of Varied Tits (*Sittiparus various*) in South Korea under future climate conditions projected under a baseline scenario (RCP 8.5; left panels) and an intermediate mitigation scenario (RCP 4.5; right panels)

**Figure 5.**
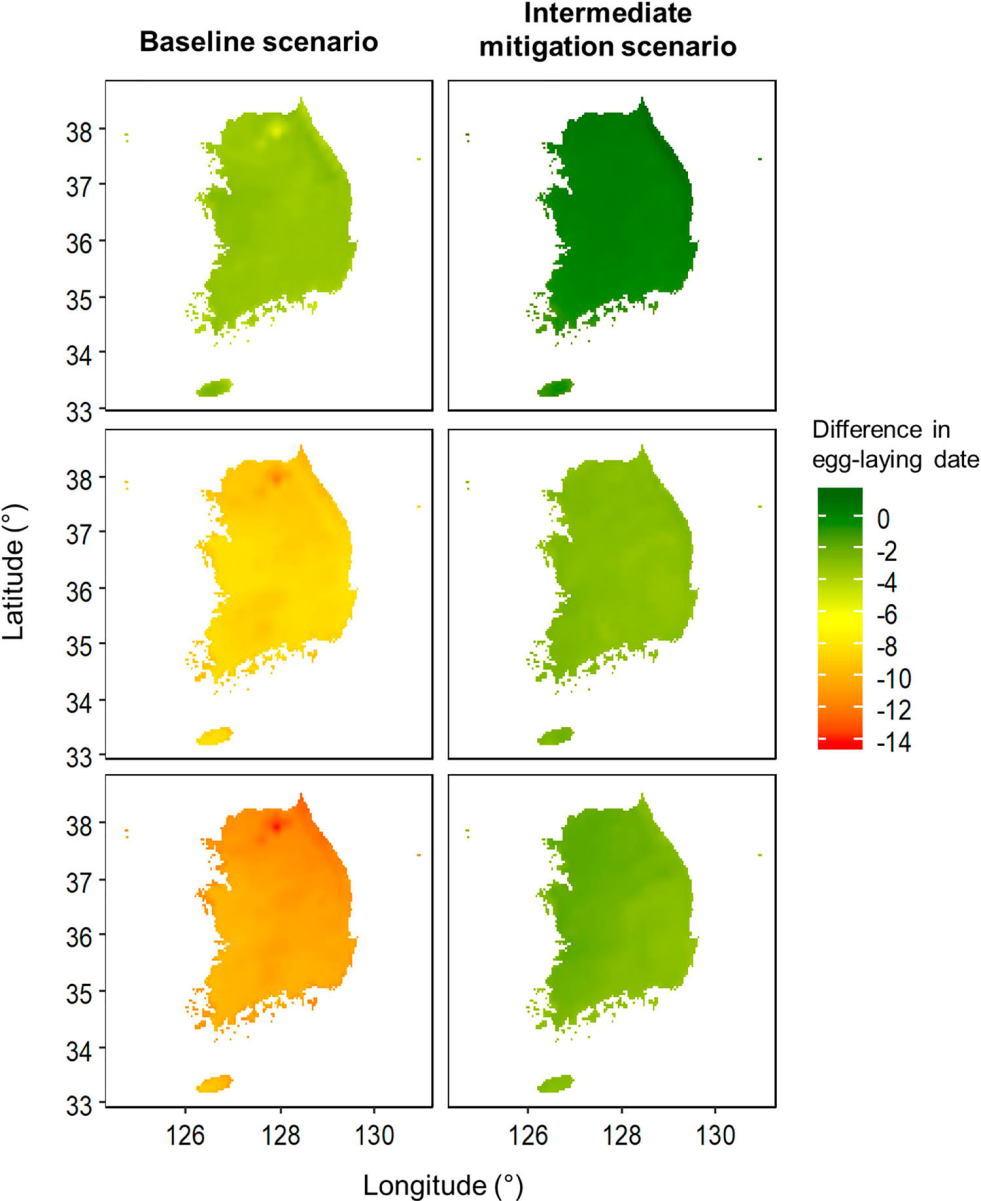
Predicted egg-laying dates of Varied Tits (*Sittiparus various*) in South Korea in the 2040s, 2070s, and 2090s compared with present dates under future climate conditions projected by a baseline scenario (RCP 8.5; left panels) and an intermediate mitigation scenario (RCP 4.5; right panels)

This model predicts earlier egg-laying under the baseline scenario than under intermediate mitigation, by 1.83 ± 0.29 days in the 2040s, 4.26 ± 0.33 days in the 2070s, and 6.64 ± 0.49 days in the 2090s (Table 3, Figure 4). Under the baseline scenario, egg-laying dates were predicted to occur earlier over time compared to the present, by 3.27 ± 0.28 days in the 2040s, 8.65 ± 0.44 days in the 2070s, and 10.63 ± 0.67 days in the 2090s (Figure 5). In 2090, the predicted egg-laying dates were more than 10 days earlier than present in 83.58% of the area in South Korea. The predicted dates were more advanced in the mountainous region of northeastern Korea compared to other regions across all periods (Figure 5).

**Table 3.** Predicted mean egg-laying dates (mean ± SD; January 1 = 1) of Varied Tits (*Sittiparus various*) in South Korea.

Scenario	Egg-laying dates			
	2011–2020	2041–2050	2071–2080	2091–2100
Baseline scenario without mitigation	110.68 ± 4.56	107.41 ± 4.60	102.03 ± 4.38	100.05 ± 4.22
Intermediate mitigation scenario	109.09 ± 4.35	109.23 ± 4.56	106.29 ± 4.36	106.70 ± 4.51

Under the intermediate mitigation scenario, there is little change predicted for the 2040s (Figure 5). In addition, the predicted dates for the 2040s were earlier by 0.33 ± 0.33 days compared to the present in 27.14% of the area in South Korea but were the same or later than present for the remaining regions (–0.32 ± 0.23 days). In the 2070s and 2090s, egg-laying dates are predicted to occur earlier across the country by up to 3.70 and 3.77 days, respectively. However, the average advancement was slightly higher in the 2070s than in the 2090s (2070s: 2.80 ± 0.17 days, 2090s: 2.39 ± 0.39 days).

## Discussion

Our results indicate that egg-laying in Varied Tits will occur earlier when the average daily mean and max temperature prior to the breeding season increases, along geographical gradients. This is in keeping with previous studies reporting that egg-laying in various temperate forest birds has occurred earlier under warming temperatures over recent decades (McCleery and Perrins 1998; Crick and Sparks 1999; Dunn and Winkler 1999; Visser et al. 2003; Both and te Marvelde 2007). The relationship we observed between temperature and breeding, combined with the warming trend of spring climate in the Korean Peninsula, predicts the same pattern observed in long-term studies conducted in other parts of the world (Dunn and Winkler 1999; Visser et al. 2003; Both et al. 2004).

The final averaged model with mean and max temperature for prediction of egg-laying dates indicates laying will take place earlier in the year according to mean temperature rather than max temperature. Our evaluation of predictive performance indicates that the bias of predication is low, and the predicted egg-laying dates are strongly correlated with the observed dates at different time scales. Also, the prediction error and unexplained variance were similar with the result of evaluation of predicted egg-laying dates in previous studies (Chmielewski et al. 2013; Gullett et al. 2013). As such, the model derived from phenological differences along geographical temperature gradients can be used as a reliable predictor for future shifts as the result of climate change. However, previous studies have found that other environmental factors such as vegetation type (e.g. deciduous vs. evergreen forest) also cause variation in phenological responses to climate change between populations (Porlier et al. 2012). Since our model was derived from a population in mixed-deciduous forests, this suggests that predictive accuracy might be lower for larger spatial scales when different habitats are included. Consideration of a broader range of habitats, sampled representatively across the range would improve the predictive accuracy of the model.

Predicted egg-laying dates of Varied Tits for the coming decades indicate that reproductive phenology will advance under both climate change scenarios, but under the baseline scenario without mitigation efforts, the shift will accelerate compared to the intermediate mitigation scenario. Egg-laying dates in the 2090s are predicted to be a mean of 10 days earlier compared to the present and will occur earlier still in the high-mountain regions of South Korea where temperatures will increase faster. Similarly, previous studies have found that temperature over recent decades has risen more quickly at higher elevations than at lower areas, with a greater predicted rate of warming (Beniston 2003; Wang et al. 2014; Pepin et al. 2015).

There is a lack of knowledge as to how the interaction between songbird and other organisms in the forest food web are affected by phenological shifts predicted by climate change in South Korea. Previous studies have shown that changes in breeding phenology can cause a mismatch between birds and their food resources (Visser et al. 1998, 2004), leading to decreased prey availability during chick-rearing periods and negatively affecting reproductive success (Thomas 2001; Sanz et al. 2003; Both et al. 2006; Visser et al. 2006). In some studies, the degree of mismatch was most severe in years with extremely high spring temperatures (Both et al. 2006; Burgess et al. 2018). Climate change models suggest an increased prevalence of extreme climatic patterns, which is likely to exacerbate the consequences of phenological mismatch described here (Both et al. 2006; Burgess et al. 2018).

Radiative forcing and global surface temperature are predicted to stabilize after the mid-twenty-first century under an intermediate mitigation scenario, but will continuously rise under baseline predictions (Riahi et al. 2011; Knutti and Sedláček 2013). As a result, egg-laying dates are likely to occur continue to occur earlier in the next century if no strategies for limiting GHG emission are adopted. However, the photoperiod cue, which controls reproductive hormone and gonadal development, is invariable among year (Dawson et al. 2001; Sharp 2005), thus they would not be able to be advanced the egg-laying dates with temperature rise earlier than a certain period when is determined by photoperiod threshold. In addition, warming temperatures may eventually exceed the limits of the thermal niche of insectivorous songbirds, which may affect their ecological traits (Jiguet et al. 2006; Khaliq et al. 2014). Numerous studies have reported that climate change has caused shifts in distribution of birds upward to higher altitudes and latitudes with lower temperature, as well as substantial reductions in range size, especially in species adapted to high-altitude environments (Hitch and Leberg 2007; Sekercioglu et al. 2008). Understanding species’ thermal limitations can also help predict responses to climate change. At present, it is not known whether many insectivorous songbirds in Korea have the flexibility to adapt to a changing climate in maintaining their current distribution.

Insectivorous songbirds in forested habitats function as the top-down controller for the density of leaf-eating caterpillar by intensive foraging, and this top-down control can reduce leaf damage and affect tree growth (Marquis and Whelan 1994). Therefore, altered phenology of insectivorous songbirds may lead to decreased tree growth and carbon fixation, further exacerbating greenhouse gas accrual. The IPCC (2018) stresses that the impacts climate change on natural ecosystems will increase if global temperature rises between 1.5°C and 2.0°C and suggests urgent mitigative action. Intensive efforts for reducing greenhouse gas emission under the Paris Agreement would help in conservation of insectivorous songbirds and forest ecosystems in South Korea. Continued long-term monitoring of multi-trophic communities, including birds and arthropod prey, should also be conducted in order to accurately assess and predict the effects of climate change on phenological shifts affecting community ecology.
